# A Systematic Review of Xuezhikang, an Extract from Red Yeast Rice, for Coronary Heart Disease Complicated by Dyslipidemia

**DOI:** 10.1155/2012/636547

**Published:** 2012-04-12

**Authors:** Qinghua Shang, Zhaolan Liu, Keji Chen, Hao Xu, Jianping Liu

**Affiliations:** ^1^Graduate School of Chinese Medicine, Beijing University, Beijing 100029, China; ^2^Centre for Evidence-Based Chinese Medicine, Beijing University of Chinese Medicine, Beijing 100029, China; ^3^Cardiovascular Disease Center, Xiyuan Hospital, China Academy of Chinese Medical Sciences, Beijing 100091, China

## Abstract

*Objective*. This systematic review aims to evaluate the benefit and side effect of Xuezhikang for coronary heart disease (CHD) complicated by dyslipidemia. *Methods*. All randomized clinical trials (RCTs) with Xuezhikang as a treatment for CHD combined with dyslipidemia were considered for inclusion. Data extraction and analyses and quality assessment were conducted according to the Cochrane standards. *Results*. We included 22 randomized trials. Xuezhikang showed significant benefit on the incidence of all-cause deaths, CHD deaths, myocardial infarction, and revascularization as compared with placebo based on conventional treatment for CHD. It remarkably lowered total cholesterol (TC), triglyceride (TG), and low-density lipoprotein-cholesterol (LDL-C) as compared with the placebo or inositol nicotinate group, which was similar to statins group. Xuezhikang also raised high-density lipoprotein cholesterol (HDL-C) compared to placebo or no intervention, which was similar to Inositol nicotinate and slightly inferior to statins. The incidence of adverse events did not differ between the Xuezhikang and control group. *Conclusions*. Xuezhikang showed a comprehensive lipid-regulating effect and was safe and effective in reducing cardiovascular events in CHD patients complicated by dyslipidemia. However, more rigorous trials with high quality are needed to give high level of evidence.

## 1. Introduction

Coronary heart disease (CHD) is one of the most serious diseases with high incidence and mortality. Dyslipidemia contributes greatly to the formation and progression of atherosclerosis (AS), which plays a dominant role in leading to CHD. Patients with CHD are also commonly complicated with dyslipidemia. Modulating dyslipidemia actively, especially lowering low-density lipoprotein-cholesterol (LDL-C) by statins, has been demonstrated to be very crucial to prevent AS and reduce the morbidity and mortality of CHD. Most recently, the updated ESC/EAS guidelines for management of dyslipidemia [1] further highlighted the aggressive lipid-lowering strategy in subjects with documented coronary vascular disease (CVD) or previous myocardial infarction (MI). However, the application of statins might be restricted by the adverse effect on the liver function and creatine kinase, especially in patients with old age, multiple comorbid diseases, high-dose statins, or a combination lipid-lowering therapy. Thus it is of great clinical significance to find an effective but safer alternative therapy in CHD patients complicated by dyslipidemia.

Xuezhikang is a partially purified extract of fermented red yeast rice (Monascus purpureus). It is composed of 13 kinds of natural statins, unsaturated fatty acids, ergosterol, amino acids, flavonoids, alkaloid, trace element, and so forth. The health enhancing qualities of this yeast have been introduced and used in China for over two thousand years. At latest systematic review indicated the beneficial effects of Xuezhikang in the treatment of hyperlipidemia [2]. Therefore, Xuezhikang has been recommended in a guideline for China adult dyslipidemia prevention [3]. Recently, clinical benefits of Xuezhikang were also found in CHD patients combined with dyslipidemia in some randomized controlled trials [4–6]. This systematic review aims to evaluate the benefit and side effect of Xuezhikang, a potential alternative drug of statins, for CHD patients complicated by dyslipidemia, and thus provide further evidence for clinical application.

## 2. Methods

### 2.1. Inclusion Criteria

Randomized controlled trials (RCTs) comparing Xuezhikang with placebo, no intervention, or established lipid-lowing agents in English or Chinese were considered. Quasirandomized trials were excluded, and the duration of the intervention was no less than four weeks. Participants of all age with CHD complicated by dyslipidemia meeting with at least one of the current or past definitions or guidelines of CHD [including acute coronary syndrome (ACS)] [7–13] and dyslipidemia (treatment goal as the lower limit, see Table 1) [14–20] were considered. Those who did not introduce diagnostic criteria in the text but stated patients with definite CHD or dyslipidemia were also included. Secondary dyslipidemia, high serum lipid level after meal, serious heart failure, and serious hepatic or renal failure were excluded.

Outcome measures include primary outcomes (including all-cause mortality, CHD mortality, incidence of MI, revascularization, and rehospitalization for unstable angina) and secondary outcomes [including serum total cholesterol (TC), triglyceride (TG), LDL-C, and-high density lipoprotein cholesterol (HDL-C)].

### 2.2. Search Strategy

Two reviewers searched the following databases up to September 2011 independently for the identifications of trials (publication or nonpublication): The Cochrane Library, Pubmed, Chinese Biomedical Database (CBM), China National Knowledge Infrastructure (CNKI), Chinese VIP Information (VIP), and Wanfang Databases. We used the terms as follows: coronary heart disease, CHD, coronary artery disease, angina pectoris, myocardial infarction, acute coronary syndrome, cardi*, and Xuezhikang, red yeast rice, monascus. Because of different characteristics of various databases, MeSH terms and free text terms were used regardless of the report types in full text, title, keyword, subject terms, or abstract.

### 2.3. Data Extraction and Quality Assessment

Two reviewers (Shang QH, Liu ZL) independently extracted data according to a data extraction form made by the authors. Disagreements were resolved by consensus or consultation from a third reviewer (Liu JP). The methodological quality of trials was assessed independently using criteria from the Cochrane Handbook for Systematic Review of Interventions, Version 5.0.1 (Shang QH, Liu ZL) [20]. We contacted with the authors if there was any doubt in randomization and blinding method. The items included random sequence generation (selection bias), allocation concealment (selection bias), blinding of participants and personnel (performance bias), blinding of outcome assessment (detection bias), incomplete outcome data (attrition bias), selective reporting (reporting bias), and other bias. We judged each item from three levels (“Yes” for a low of bias, “No” for a high risk of bias, “Unclear” otherwise), and then we assessed the trials and categorized them into three levels: low risk of bias (all the items were in low risk of bias), high risk of bias (at least one item was in high risk of bias), unclear risk of bias (at least one item was in unclear).

### 2.4. Data Synthesis

We used Revman 5.1 software provided by the Cochrane Collaboration for data analyses. Studies were stratified by the types of comparisons. We will express dichotomous data as risk ratio (RR) and its 95% confidence intervals (CI). Continuous outcome will be presented as mean difference (MD) and its 95% CI. Heterogeneity was recognized significant when *I* ^2^≧50%. Fixed effects model was used if there is no significant heterogeneity of the data; random effects model was used if significant heterogeneity existed (50% < *I* ^2^ < 85%). Publication bias was explored using a funnel plot.

## 3. Results

### 3.1. Description of Included Trials

**Figure 1:**
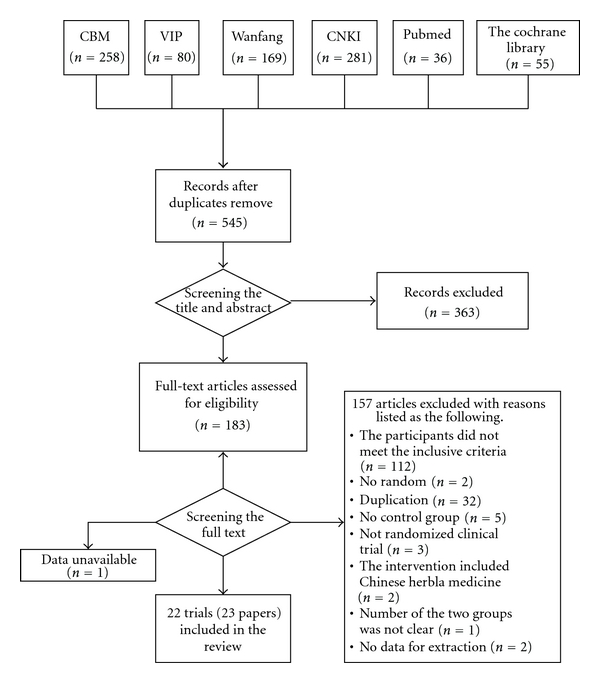
Flow chart of study selection.

22 RCTs (23 papers) [4–6, 21–39] were included, 21 papers were published in Chinese, one paper published in English, and one was unpublished as a postgraduate dissertation. The whole process of trials selection was demonstrated in Figure 1. The characteristics of included trials were listed in Table 2.

6520 Participants were included (3264 in intervention group and 3256 in control group). Two of the trials did not report the gender, and 4905 male and 1538 female were included in the other 20 trials. A total of 7 criteria of CHD (including ACS) were selected, but 6 trials did not introduce criteria of CHD but mentioned “patients with CHD were eligible to include.” 3 criteria of dyslipidemia were used for 11 trials, and the other 11 trials only reported the serum lipid levels, which were categorized to dyslipidemia according to the previous and current definitions and guidelines Table 1. One trial [4] included patients with MI; five of the trials [5, 27, 28, 34, 39] included patients with unstable angina; two of the trials [6, 38] included patients with ACS; three of the trials [21, 22, 31] included patients with stable angina. The other 11 trials [23–26, 29, 30, 32, 33, 35–37] did not introduce the types of CHD or all types were included.

Patients in 19 trials prescribed Xuezhikang 600 mg QD (regulation was conducted for adverse events), one trial used Xuezhikang 600 mg TID if the serum TC or TG still higher after having been prescribed for 6 weeks (600 mg BID in previous 6 weeks) [30], one trial [37] prescribed Xuezhikang 300 mg TID, and one trial [31] prescribed Xuezhikang 1200 mg QN. The duration of treatment ranged from 4 weeks to 7 years.

There were five comparisons in the review according to various control groups. (1) Xuezhikang and conventional therapy versus conventional therapy (8 trials) [5, 6, 24, 29, 33, 34, 38, 39]; (2) Xuezhikang and conventional therapy versus placebo and conventional therapy (2 trials) [4, 35]; (3) Xuezhikang and conventional therapy versus statin and conventional therapy (9 trials) [21–23, 25, 26, 28, 31, 37, 39]; (4) Xuezhikang and statin and conventional therapy versus statin and conventional therapy (2 trials) [27, 36]; (5) Xuezhikang and aspirin versus inositol nicotinate and aspirin (1 trials) [32]. One trial [39] was designed as three groups with two comparisons and Xuezhikang and conventional therapy versus conventional therapy; Xuezhikang and conventional therapy versus atorvastatin and conventional therapy.

### 3.2. Methodological Quality of Included Trials

**Figure 2:**
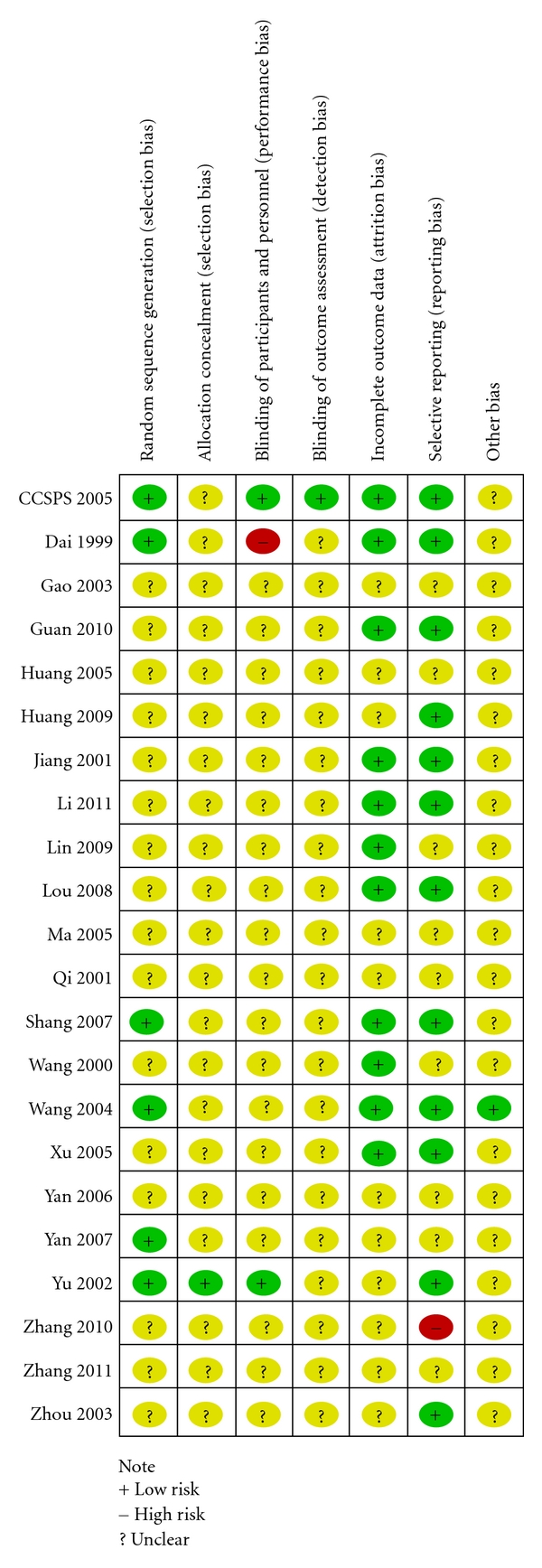
Risk of bias summary.

According to the criteria introduced above, no trial was evaluated as having a low risk of bias. Only one trial of the 22 trials reported the method to generate the allocation sequence (random number table) in the paper [6]. After we contacted with the authors, six trials announced a correct method for allocation sequence [4–6, 31, 33, 35]. One trial was assessed as having adequate concealment [35]. Two trials applied double-blinding [4, 35], and two trials used single-blinding but did give us objective to be blinded [25, 37]. One trial blinded the outcome assessors [4]. One trial reported prior sample size estimation and mentioned intention-to-treat analysis [4]. Five trials reported information on withdrawal/dropout [4, 6, 22, 29, 32]. 18 trials [4–6, 22–27, 29, 31–33, 35–39] provided baseline data for the comparability among groups. The results of the assessment of risk of bias are presented in a “risk of bias summary” figure produced by Revman 5.1 automatically Figure 2.

### 3.3. Effect Estimates of Outcomes (Tables 3 and 4)

#### 3.3.1. All-Cause Mortality

There was only 1 trial [4] reported the all-cause mortality in the comparisons of Xuezhikang and conventional therapy versus placebo and conventional therapy [RR 0.67; 95% CI 0.54 to 0.83; 1 trial, *n* = 4870].

#### 3.3.2. Mortality of CHD

There were 5 studies [4, 22, 27, 28, 32] that presented the effect of Xuezhikang in reducing the mortality of CHD. Compared to placebo on the basis of conventional therapy, Xuezhikang showed a reduction of mortality of CHD (RR 0.69; 95% CI 0.54 to 0.89; 1 trial, *n* = 4870) [4]. Compared to simvastatin on the basis of conventional therapy, Xuezhikang showed no significant difference in mortality of CHD (RR 0.26; 95% CI 0.06 to 1.21; 2 trial, *n* = 220) [22, 28]. Compared to no treatment on the basis of simvastatin and conventional therapy, Xuezhikang showed no effect in reducing mortality of CHD (RR 0.33; 95% CI 0.01 to 7.80; 1 trial, *n* = 48) [27]. Compared with inositol nicotinate on the basis of aspirin, Xuezhikang showed no significant difference in mortality of CHD (RR 0.15; 95% CI 0.02 to 1.18; 1 trial, *n* = 122) [32].

#### 3.3.3. Incidence of MI

There were 3 studies reporting CHD events in 3 different comparisons. Compared with placebo on the basis of conventional therapy, Xuezhikang showed a reduction of morbidity of MI (RR 0.39; 95% CI 0.28 to 0.55; 1 trial, *n* = 4870) [4]. Compared with simvastatin on the basis of conventional therapy, Xuezhikang showed no significant difference (RR 0.95; 95% CI 0.30 to 3.05; 1 trial, *n* = 84) [28]. In comparisons of Xuezhikang and simvastatin and conventional therapy versus simvastatin and conventional therapy, Xuezhikang showed no effect in reducing incidence of MI (RR 0.20; 95% CI 0.01 to 3.96; 1 trial, *n* = 48) [27].

#### 3.3.4. Revascularization

Revascularization included percutaneous coronary intervention (PCI) and coronary artery bypass graft (CABG). There were 2 studies [4, 28] reporting revascularization in 2 different comparisons. Compared with placebo on the basis of conventional therapy, Xuezhikang showed a significant reduction of revascularization (RR 0.67; 95% CI 0.50 to 0.89; 1 trial, *n* = 4870) [4]. Compared with simvastatin on the basis of conventional therapy, Xuezhikang showed no significant difference (RR 1.14; 95% CI 0.38 to 3.46; 1 trial, *n* = 84) [28].

#### 3.3.5. Rehospitalization for Unstable Angina

There were 2 trials [27, 28] reporting rehospitalization in 2 different comparisons. Compared with simvastatin on the basis of conventional therapy, Xuezhikang showed no significant difference in the number of rehospitalization (RR 1.02; 95% CI 0.57 to 1.84; 1 trial, *n* = 84) [28]. Compared with no treatment on the basis of simvastatin and conventional therapy, Xuezhikang showed no effect in reducing rehospitalization (RR 0.20; 95% CI 0.03 to 1.59; 1 trial, *n* = 48) [27].

#### 3.3.6. Serum TC Level

There were 21 studies that reported the level of total cholesterol Table 4, but one trial only reported the serum lipid level of the treatment group [30]. (1) Compared to no treatment with cointervention of conventional therapy, Xuezhikang showed a reduction of TC level (MD −0.97 mmol/L; 95% CI −1.24 to −0.71; 8 trials, *n* = 500) [5, 6, 24, 29, 33, 34, 38, 39]. (2) There were two trials that reported Xuezhikang versus placebo on the basis of conventional therapy, meta-analysis was not used for significant difference, and, in this comparison, Xuezhikang showed a reduction of TC level (MD −0.57 mmol/L; 95% CI −0.61 to −0.53; 1 trial, *n* = 4870) [4] and (MD −2.62 mmol/L; 95% CI −2.98 to −2.26; 1 trial, *n* = 62) [35]. (3) There was no significant difference on serum TC level of Xuezhikang comparing to statins on the basis of conventional therapy (MD 0.19 mmol/L; 95% CI −0.22 to 0.59; 8 trial, *n* = 633) [21, 23–25, 28, 31, 37, 39]. Since there was significant heterogeneity in the comparison, we examined the data carefully and found that data of two trials deviated from the others. After looking over the papers, one of the two trial [26] with an unclear conventional therapy and the other used Xuezhikang 300 mg tid in the whole trial [37]. Sensitive analysis was used and got a similar conclusion (MD 0.02 mmol/L; 95% CI −0.03 to 0.06; 6 trial, *n* = 489) after excluded the two trials [26, 37]. (4) Compared with no treatment on the basis of statins and conventional therapy, Xuezhikang showed a reduction of TC level (MD −0.96 mmol/L; 95% CI −1.33 to −0.58; 2 trial, *n* = 108) [27, 36]. (5) Compared to inositol nicotinate on the basis of aspirin, Xuezhikang showed a significant difference in the reduction of TC level (MD −1.05 mmol/L; 95% CI −1.46 to −0.64; 1 trial, *n* = 105) [32].

#### 3.3.7. Serum TG Level

There were 20 studies that reported the level of TG (See Table 4), but one trial only reported the serum lipid level of the treatment group [30]. (1) Compared to no treatment with cointervention of conventional therapy, Xuezhikang showed a reduction of TG level (MD −0.49 mmol/L; 95% CI −0.58 to −0.39; 7 trial, *n* = 412) [5, 6, 24, 29, 33, 38, 39]. (2) There were two trials that reported Xuezhikang versus placebo on the basis of conventional therapy, meta-analysis was not used for significant difference, and, in this comparison, Xuezhikang showed a reduction of TG level (MD −0.17 mmol/L; 95% CI −0.22 to −0.12; 1 trial, *n* = 4870) [4] and (MD −1.29 mmol/L; 95% CI −1.57 to −1.01; 1 trial, *n* = 62) [35]. (3) There was no significant difference on serum TG level of Xuezhikang comparing to statins on the basis of conventional therapy (MD −0.05 mmol/L; 95% CI −0.12 to 0.02; 8 trial, *n* = 633) [21, 23–25, 28, 31, 37, 39]. (4) Compared with no treatment on the basis of fluvastatin and conventional therapy, Xuezhikang showed a reduction of TG level (MD −0.27 mmol/L; 95% CI −0.35 to −0.19; 1 trial, *n* = 60) [36]. (5) Compared to inositol nicotinate on the basis of aspirin, Xuezhikang showed a significant difference in the reduction of TG level (MD −0.60 mmol/L; 95% CI −0.95 to −0.25; 1 trial, *n* = 105) [32].

#### 3.3.8. Serum LDL-C Level

There were 21 studies that reported the level of LDL-C (see Table 4), but one trial only reported the serum lipid level of the treatment group [30]. (1) Compared to no treatment with cointervention of conventional therapy, Xuezhikang showed a reduction of LDL-C level (MD −0.78 mmol/L; 95% CI −1.19 to −0.38; 7 trial, *n* = 444) [5, 6, 24, 33, 34, 38, 39]. (2) There were two trials that reported Xuezhikang versus placebo on the basis of conventional therapy, meta-analysis was not used for significant difference, and, in this comparison, Xuezhikang showed a reduction of LDL-C level (MD −0.57 mmol/L; 95% CI −0.62 to −0.52; 1 trial, *n* = 4870) [4] and (MD −1.82 mmol/L; 95% CI −2.01 to −1.63; 1 trial, *n* = 62) [35]. (3) There was no significant difference on serum LDL-C level of Xuezhikang comparing to statins on the basis of conventional therapy (MD 0.03 mmol/L; 95% CI −0.10 to 0.25; 8 trial, *n* = 633) [21, 23–25, 28, 31, 37, 39]. Because there was significant heterogeneity in the comparison, we examined the data carefully and found that data of two trials deviated from the others. After looking over the papers, one of the two trials [26] with an unclear conventional therapy and the other used Xuezhikang 300 mg tid in the whole trial [37]. Sensitive analysis was used and got a similar conclusion (MD 0.05 mmol/L; 95% CI −0.09 to 0.19; 6 trial, *n* = 489) after excluded the two trials [26, 37]. (4) Compared with no treatment on the basis of statins and conventional therapy, Xuezhikang showed a reduction of LDL-C level (MD −0.44 mmol/L; 95% CI −0.57 to −0.31; 2 trial, *n* = 108) [27, 36]. (5) Compared to inositol nicotinate on the basis of aspirin, Xuezhikang showed a significant difference in the reduction of LDL-C level (MD −0.88 mmol/L; 95% CI −1.27 to −0.48; 1 trial, *n* = 105) [32].

#### 3.3.9. Serum HDL-C Level

There were 19 studies that reported the level of HDL-C (See Table 4), but one trial only reported the serum lipid level of the treatment group [30]. (1) Compared to no treatment with cointervention of conventional therapy, Xuezhikang showed a beneficial effect of HDL-C level (MD 0.24 mmol/L; 95% CI 0.08 to 0.40; 6 trial, *n* = 364) [5, 6, 24, 33, 34, 39]. (2) There were two trials that reported Xuezhikang versus placebo on the basis of conventional therapy, meta-analysis was not used for significant difference, and, in this comparison, Xuezhikang showed a beneficial effect of HDL-C level (MD 0.05 mmol/L; 95% CI 0.03 to 0.07; 1 trial, *n* = 4870) [4] and (MD 0.48 mmol/L; 95% CI 0.37 to 0.59; 1 trial, *n* = 62) [35]. (3) There was a lower effect on serum HDL-C level of Xuezhikang comparing to statins on the basis of conventional therapy (MD −0.10 mmol/L; 95% CI −0.19 to −0.01; 8 trial, *n* = 633) [21, 23–25, 28, 31, 37, 39]. Because there was significant heterogeneity in the comparison, we examined the data carefully and found that data of one trials deviated from the others. After looking over the papers, we found that the trial used Xuezhikang 300 mg tid [37]. Sensitive analysis was used and got a similar conclusion (MD −0.10 mmol/L; 95% CI −0.11 to −0.08; 7 trial, *n* = 553) after excluded the trial [37]. (4) Compared with no treatment on the basis of fluvastatin and conventional therapy, Xuezhikang showed a beneficial of HDL-C level (MD 0.15 mmol/L; 95% CI 0.05 to 0.25; 1 trial, *n* = 60) [36]. (5) Compared with inositol nicotinate on the basis of aspirin, Xuezhikang showed no significant difference on HDL-C level (MD 0.17 mmol/L; 95% CI −0.21 to 0.55; 1 trial, *n* = 105) [32].

### 3.4. Publication Bias

**Figure 3:**
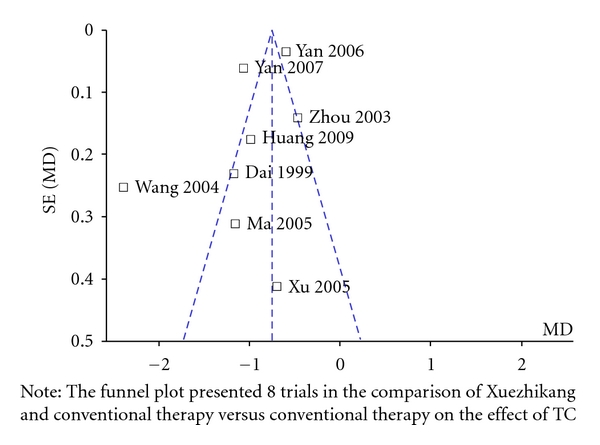
The funnel plot for assessing reporting bias.

A funnel plot analysis of the 8 trials in comparison of Xuezhikang and conventional therapy versus conventional therapy on serum TC level was conducted and shown in Figure 3.

### 3.5. Adverse Events

There were 17 trials that reported adverse events (Ads); see Table 5. 4 of the 17 trials [5, 24, 33, 37] indicated no Ads in the duration of treatment, and 2 trials [23, 34] only introduced that there was no difference of the two groups. The most commonly reported Ads in the 10 trials were intestinal disturbance (abdominal distension, constipation, and diarrhea), dizziness, high serum alanine aminotransferase (ALT), high serum creatine kinase (CK), high serum creatinine, high blood urea nitrogen (BUN), and skin itch. All of Ads were not significantly different between the Xuezhikang group and control group. One trial [4] reported that there was significant difference between the two groups on sexual dysfunction (*P* = 0.0253) in the paper, but after we import the data into Revman 5.1, there was no difference (RR 0.09, 95% CI [0.01,1.64]) between the two groups. CCSPS [4] reported the clinical total Ads number (intestinal disturbance, allergy and et al.) in each group (treatment group 43; control group 39), and there was no significant difference between the two groups, this trial also reported death in other reason, which was introduced in all-cause mortality, and the difference between the two groups was not significant.

## 4. Discussion

This systematic review included 22 randomized trials and a total of 6520 participants. Xuezhikang showed significant benefit on the incidence of all-cause deaths, CHD deaths, myocardial infarction, and revascularization as compared with placebo or no intervention based on conventional treatment for CHD. It remarkably lowered TC, TG, and LDL-C as compared with the placebo or inositol nicotinate group, which was similar to statins group. Xuezhikang also significantly raised HDL-C compared to placebo or no intervention, which was similar to inositol nicotinate and slightly inferior to statins. The incidence of adverse events did not differ between the Xuezhikang and control group. The results showed the comprehensive lipid-regulating effect of Xuezhikang and indicated that it was safe and effective in reducing cardiovascular events in CHD patients complicated by dyslipidemia.

Due to the potential side effects of statins, natural products have raised more and more attention worldwide. The health-enhancing qualities of red yeast rice have been introduced and used in China for over two thousand years. A meta-analysis of randomized controlled trials on Chinese red yeast rice for primary hyperlipidemia showed a significant reduction in serum levels of TC, TG, LDL-C, and an increase in HDL-C levels compared with placebo. The lipid modification effects appeared to be similar to pravastatin, simvastatin, atorvastatin, lovastatin, or fluvastatin [40]. A latest systematic review also indicated the beneficial effects of Xuezhikang in the treatment of hyperlipidemia [2]. The lipid-regulating effects of Xuezhikang in these reviews were similar to our findings. In addition, some cardioprotective effects of Xuezhikang have been investigated in recent years [41–43]. We further demonstrated the benefit of Xuezhikang in reducing cardiovascular events in CHD patients complicated by dyslipidemia, or even CHD with normal blood lipid level but failed to reach the lipid-lowering goal. However, current evidence comparing the effectiveness and Ads between Xuezhikang and statins in CHD patients was not enough to draw the conclusion.

It is worth mentioning China Coronary Secondary Prevention Study (CCSPS) [4], which was the largest RCT included in this review. This multicenter, randomized, and placebo-controlled study aimed to demonstrate the long-term therapeutic effect and safety of Xuezhikang in the second prevention of CHD. 4870 cases in 66 medical centers were enrolled and followed up for an average of 4.5 years. The results showed that Xuezhikang significantly decreased the recurrence of coronary events and the occurrence of new cardiovascular events and deaths, improved lipoprotein regulation, and was safe and well tolerated [4]. The study was the first large-scale clinical trial in eastern population who suffered from mild or moderate degree of hyperlipidemia and previous MI. The CCSPS study is quite comparable with (Cholesterol and Recurrent Events) CAREs study [44] in terms of the target population, sample size, baseline lipid and follow-up time. However, Xuezhikang in CCSPS lowered less lipid level as compared with pravastatin in CARE but seemed to gain more benefit in reducing the cardiovascular events. Since the effect of Xuezhikang is partially attributed to the presence of statins, it has been hypothesized that relatively high concentrations of unsaturated fatty acids and other natural compounds found in Xuezhikang may work in concert with the statins to provide additional health benefits [45]. Therefore, a large-scale RCT comparing directly the effectiveness and safety of long-term use of Xuezhikang and statins is warranted.

Before recommending the conclusion of this review to clinical practicers, we have to consider the following weaknesses in this review. (1) Firstly, the “randomization” was not clear in most of the trials for insufficient reporting of generation methods of the allocation sequence and allocation concealment. Most trials stated only that patients were randomly assigned. (2) Secondly, most of trials did not introduce double blind in this review, and only one trial introduced blinding of outcome assessment, therefore, in non-placebo-controlled and non-double-blind trials, placebo effects may add to the complexity of interpreting the conclusion. (3) Most of the trials did not introduce the study plan, attrition bias and selective reporting bias might exist in this conclusion. (4) Thirdly, funnel plot indicated that publication bias would exist in this review. The reasons are as follows. We only selected trials published in Chinese and English, and trials published in other language or originated from other countries might be omitted; we only identified unpublished studies from conference paper or academic thesis, and negative trials might not be reported and induced publication bias.

Therefore, further rigorously designed trials are still needed before Xuezhikang could be recommended to patients with CHD complicated by dyslipidemia, especially as an alternative to statins. Whether or not long-term medication of Xuezhikang could provide similar benefit to statins for CHD secondary prevention with less adverse events? Is it related to the target lipid value? All of these need to be answered in the future investigation.

## 5. Conclusion

Xuezhikang showed a comprehensive lipid-regulating effect and was safe and effective in reducing CHD mortality, the incidence of myocardial infarction and revascularization in CHD patients complicated by dyslipidemia. However, the small sample size and potential bias of most trials influence the convincingness of this conclusion. Before recommending Xuezhikang as an alternative to statins in CHD patients, more rigorous trials with high quality are needed to give high level of evidence, especially for comparing the effectiveness and safety between Xuezhikang and statins.

## Figures and Tables

**Table 1:** Definition of dyslipidemia or treatment goal of patients with CHD or equivalents on serum lipid level.

Origination	Definition of dyslipidemia or treatment goal of Patients with CHD or equivalents on serum lipid level
ATP I 1988 [14]	Ideal lipid level: TC < 5.17 mmol/L (200 mg/dL); LDL-C < 3.36 mmol/L (130 mg/dL). Patients with HDL-C < 0.9 mmol/L (35 mg/dL) were defined unmoral. The definition of dyslipidemia was according to the level of LDL-C

ATP II 1993 [15]	Treatment goal: LDL-C≦2.6 mmol/L (100 mg/dL)

Ministry of Health of the People's Republic of China 1993 [8]	The treatment goal was not introduced

CADPS 1997 [16]	Treatment goal: TC < 4.68 mmol/L (180 mg/dL); TG < 1.7 mmol/L (150 mg/dL); LDL-C < 2.6 mmol/L (100 mmol /L)

ATP III 2001 [17]	Treatment goal: LDL-C < 2.6 mmol/L (100 mg/dL)

Implication of ATP III 2004 [18]	Treatment goal: LDL-C < 2.6 mmol/L (100 mg/dL); the optional goal: LDL-C < 1.8 mmol/L (70 mg/dL)

AHA/ACC Guideline 2006 [19]	Treatment goal: LDL-C < 2.6 mmol/L (100 mmol/L), and it is seasonal for lower than 1.8 mmol/L (70 mg/dL)

CADPG 2007 [3]	Treatment goal: TC < 4.14 mmol/L (160 mg/dL) and LDL-C < 2.59 mmol/L (100 mg/dL) for CHD or equivalents Treatment goal: TC < 3.11 mmol/L (120 mg/dL); LDL-C < 2.07 mmol/L (80 mg/dL) for ACS or ischemic cardiovascular disease complicated with diabetes mellitus Suitable scope of HDL-C: *⩾*1.04 mmol/L (40 mg/dL); suitable scope of TG: <1.7 mmol/L (150 mg/dL)

ESC/EAS 2011 [1]	In patients at very high CV risk (established CVD, type 2 diabetes, type I diabetes with target organ damage, moderate to severe CKD or a score level≧10%), the LDL-C goal is <1.8 mmol/L (70 mg/dL) and/or *⩾*50% LDL-C reduction when target level cannot be reached (I A recommendation)

**Table 2:** Characteristics of included trials.

ID	Diagnostic criteria of CHD (ACS)	Diagnostic criteria of dyslipidemia	Types of CHD	Sample size (I/C)	Age (y, I/C)	Interventions group	Control group	Duration of treatment	Outcomes evaluation	Balance report of baseline
CCSPS 2005 [4]	Not specified	TC: 4.40–6.47	MI	2441/2429	(Male: 58.1 ± 9.9; female: 62.9 ± 6.7) /(male: 58.0 ± 9.7; female: 62.6 ± 7.4)	Xuezhikang 600 mg BID + conventional therapy (no detail)	Placebo + conventional therapy (no detail)	4 year in average	Serum lipid level (TC, TG, LDL-C, HDL-C), all-cause mortality, cardiovascular events, serum lipid level (TC, TG, HDL-C, LDL-C), ADs	Yes

Dai et al. 1999 [5]	WHO 1979 and Gao 1994	Ministry of Health of the People's Republic of China 1993	Unstable angina	33/25	(57 ± 9)/(56 ± 8)	Xuezhikang 600 mg, BID + control	Nitrate esters 10 mg BID + nifedipine GIFTS 30 mg QD/diltiazem 30 mg tid + metoprolol 12.5 mg BID + aspirin 50 mg QD	8 weeks	Serum lipid level (TC, TG, HDL-C, LDL-C), ADs	Yes

Gao and Liao 2003 [21]	Not specified	TC ≥ 5.2 mmol/L, LDL-C ≥ 3.12 mmol/L, TG ≥ 1.7 mmol/L	Stable Angina	30/30	53–85, 67.5 in average	Xuezhikang 600 mg BID + conventional therapy (no detail)	Fluvastatin (Lescol see fluvastatin) 20 mg QD + conventional therapy (no detail)	4 weeks	Serum lipid level (TC, TG, LDL-C, HDL-C)	Unclear

Guan 2010 [22]	Not specified	TC > 7.08 mmol/L; TG > 3.34; LDL-C > 4.2; HDL < 0.93. Two items of the above were included	Stable Angina	72/64	49–76, 62 in average	Xuezhikang 600 mg BID	Simvastatin 10 mg QN	1 year	CHD mortality, ADs	Yes

Huang et al. 2005 [23]	WHO 1979	CADPS 1997	OMI and UA	45/63	44–72	Xuezhikang 600 mg BID	Simvastatin 20 mg QN	6 weeks	Serum lipid level (TC, TG, LDL-C, HDL-C)	Yes

Huang et al. 2009 [24]	WHO 1979	CADPS 1997	Unclear	43/42	65.78 ± 4.62	Xuezhikang 600 mg, BID + control	Nitroglycerine 20 mg BIDIV + 10% KCL + insulinIV QD	12 weeks	Serum lipid level (TC, TG, HDL-C, LDL-C)	Yes

Jiang and Cai 2001 [25]	Not specified	CADPS 1997	Unclear	30/45	51 ± 8	Xuezhikang 600 mg BID + conventional therapy (as same as B)	Simvastatin 10 mg QN + conventional therapy (nitrate esters 10 mg tid, aspirin 100 mg QD or anticoagulation drugs or thrombolytic drug or hypoglycemic)	8 weeks	Serum lipid level (TC, TG, LDL-C, HDL-C), ADs	Yes

Li et al. 2011 [26]	References [12, 13]	As same as Guan 2010	Unclear	32/32	(46.9 ± 14.5) /(50.7 ± 15.1)	Xuezhikang 600 mg BID	Lovastatin 40 mg QD (20 mg QD if the ALT or AST was 3 times higher than the normal)	8 weeks	Serum lipid level (TC, TG, LDL-C, HDL-C), ADs	Yes

Lin et al. 2009 [27]	Chinese Society of cardiology 2000	TC ≥ 4.68 mmol/L or LDL-C ≥ 2.6 mmol/L	Unstable angina	24/24	35–71, 55.4 in average	Xuezhikang 600 mg, BID + control	Simvastatin 60 mg QN + conventional therapy (nitrate esters, *β* adrenergic blocking agent, CCB, aspirin, low molecular heparin and et al.	6 months	Serum lipid level (TC, LDL-C), CHD events	Yes

Lou et al. 2008 [28]	Chinese society of cardiology 2000	TC > 3.64 mmol/L and TG > 3.9 mmol/L and LDL-C > 2.6	Unstable angina	43/41	65 ± 10	Xuezhikang 600 mg BID + conventional therapy (as same as B)	Simvastatin 20 mg QD + conventional therapy (anticoagulation drugs, nitrate esters, *β* adrenergic blocking agent, ACEI, CCB and et al.)	6 months	Serum lipid level (TC, TG, LDL-C, HDL-C), Cardiovascular events, ADs	Unclear

Ma and Teng 2005 [29]	WHO 1979	CADPS 1997	Unclear	29/28	(62.7 ± 6.5) /(61.2 ± 7.1)	Xuezhikang 600 mg BID + control	Conventional therapy (nitrate esters, *β* adrenergic blocking agent, ACEI, CCB and et al.)	8 weeks	Serum lipid level (TC, TG)	Yes

Qi et al. 2001 [30]	WHO 1979	TC > 6.0 mmol/L	Unclear	60/60	60.6 ± 12.3	Xuezhikang 600 mg, BID (600 mg TID if the lipid level was still higher than the treatment goal) + control	Conventional therapy (nitrate esters, *β* adrenergic blocking agent, ACEI, CCB, and et al.)	12 weeks	Serum lipid level (TC, TG), ADs	Unclear

Shang 2007 [31]	WHO 1979	CADPS 1997	Stable Angina	65/65	(51 ± 10)/(55 ± 10)	Xuezhikang 1200 mg QN + conventional therapy (as same as control group)	Atorvastatin 10 mg QN + conventional therapy (aspirin, nitrate esters, *β* adrenergic blocking agent, ACEI, and et al.)	2 months	Serum lipid level (TC, TG, LDL-C, HDL-C)	Yes

Wang and Xiao 2000 [32]	WHO 1979	CADPS 1997	MI, UA, CHD with no symptoms	65/57	49–76, 62 in average	Xuezhikang 600 mg BID + aspirin 50 mg QD	Inositol niacinate 400 mg TID + aspirin 50 mg QD	1 year	Serum lipid level (TC, TG, LDL-C, HDL-C), cardiovascular evnets, ADs	Yes

Wang et al. 2004 [6]	ACC/AHH 2000	CADPS 1997	ACS	26/26	(60.1 ± 8.9) /(59.7 ± 8.6)	Xuezhikang 600 mg BID + control	Conventional therapy (aspirin, nitrate esters, *β* adrenergic blocking agent, ACEI, and et al.)	12 weeks	Serum lipid level (TC, TG, LDL-C, HDL-C), ADs	Yes

Xu 2005 [39]	Chinese Society of cardiology 2000	Not specified	UA	12/13/10	Unclear	Xuezhikang 600 mg BID + control group (1)	(1) Conventional therapy (isosorbide dinitrate 10 mg tid, betaloc 25–50 mg BID/TID, aspirin 50–150 mg QD, low molecular heparin 0.4–0.6 mL Q12H or diltiazem 30 mgtid/qid, or plendil 5 mg QD/BID or captopril 12.5–25 mg TID or nitroglycerine) (2) Conventional therapy (as same as (1)) and atorvastatin 20 mg Qn	1 month	Serum lipid level (TC, TG, LDL-C, HDL-C)	Yes

Yan 2006 [34]	Chinese Society of cardiology 2000	LDL-C: 1.84–4.12 mmol/L	UA	44/44	56.8 ± 8.6	Xuezhikang 600 mg BID + control	magnesium polarizing liquorIV + heparinIH + Aspirin, Nitrate esters, *β* adrenergic blocking agent CCB and et al.	8 weeks	Serum lipid level (TC, TG, LDL-C, HDL-C), ADs	Unclear

Yan and Li 2007 [33]	WHO 1979	CADPS 1997	Unclear	28/28	(66.68 ± 4.23) /(66.79 ± 4.48)	Xuezhikang 600 mg, BID + control	Nitroglycerine 20 mg BID.iv + 10% KCL + insulinIV QD	8 weeks	Serum lipid level (TC, TG, LDL-C, HDL-C)	Yes

Yu et al. 2002 [35]	WHO 1979	CADPS 1997	Unclear	32/30	(53.5 ± 10.8) /(50.6 ± 6.7)	Xuezhikang 600 mg, BID + conventional therapy (as same as control)	Placebo + conventional therapy (aspirin, nitrate esters, CCB and et al.)	8 weeks	Serum lipid level (TC, TG, LDL-C, HDL-C)	Yes

Zhang 2010 [36]	Reference [8]	CADPS 1997	Unclear	30/30	(58–80, 72.3 in average) /(59–82, 73.1 in average)	Xuezhikang 600 mg, BID + control	Fluvastatin 40 mg QD	4 weeks	Serum lipid level (TC, TG, LDL-C, HDL-C)	Yes

Zhang 2011 [37]	Unclear	CHOL > 5.72 mmol/L or LDL-C > 3.64 mmol/L complicated with high TG level	Unclear	40/40	(50 ± 13) /(45 ± 15)	Xuezhikang 300 mg TID	Atorvastatin 20 mg/d QD	8 weeks	Serum lipid level (TC, TG, LDL-C), ADs	Yes

Zhou et al. 2003 [38]	Unclear	TC > 6.0 mmol/L and (or) LDL-C > 4.2 mmol/L or complicate with >1.92 mmol/L	ACS		60.8 ± 10.6	Xuezhikang 600 mg BID + control	Conventional therapy (nitrate esters, *β* adrenergic blocking agent, CCB, anticoagulation drugs, thrombolytic drug, PTCA and et al.)	8 weeks	Serum lipid level (TC, TG, LDL-C)	Yes

**Table 3:** Analysis of clinical events.

Outcomes (comparisons)	Treatment group (*n/N*)	Control group (*n/N*)	RR	95% CI
*(1) All-cause mortality*				
Xuezhikang capsule and conventional therapy versus placebo and conventional therapy				
CCSPS 2005 [4]	126/2429	189/2441	0.67	[0.54,0.83]
*(2) Mortality of CHD*				
(2.1) Xuezhikang capsule and conventional therapy versus placebo and conventional therapy				
CCSPS 2005 [4]	92/2429	134/2441	0.69	[0.54,0.89]
(2.2) Xuezhikang and conventional therapy versus simvastatin and conventional therapy				
Guan 2010 [22]	1/72	6/64	0.15	[0.02,1.20]
Lou et al. 2008 [28]	1/43	1/41	0.95	[0.06,14.75]
	**Overall (FEM, ** *I^2^* = 13%**) **		**0.26**	[0.06,1.21]
(2.3) Xuezhikang and simvastatin and conventional therapy versus simvastatin and conventional therapy				
Lin et al. 2009 [27]	0/24	1/24	0.33	[0.01,7.8]
(2.4) Xuezhikang and aspirin versus inositol nicotinate and aspirin				
Wang and Xiao 2000 [32]	1/65	6/57	0.15	[0.02,1.18]
*(3) Myocardial infarction*				
(3.1) Xuezhikang and conventional therapy versus placebo and conventional therapy				
CCSPS 2005 [4]	47/2429	120/2441	0.39	[0.28,0.55]
(3.2) Xuezhikang and conventional therapy versus simvastatin and conventional therapy				
Lou et al. 2008 [28]	5/43	5/41	0.95	[0.30,3.05]
(3.3) Xuezhikang and simvastatin and conventional therapy versus simvastatin and conventional therapy				
Lin et al. 2009 [27]	0/24	2/24	0.2	[0.01,3.96]
*(4) Revascularization*				
(4.1) Xuezhikang capsule and conventional therapy versus placebo and conventional therapy				
CCSPS 2005 [4]	73/2429	110/2441	0.67	[0.50,0.895]
(4.2) Xuezhikang and conventional therapy versus simvastatin and conventional therapy				
Lou et al. 2008 [28]	6/43	5/41	1.14	[0.38,3.46]
*(5) Rehospitalization*				
(5.1) Xuezhikang and conventional therapy versus simvastatin and conventional therapy				
Lou et al 2008 [28]	15/43	14/41	1.02	[0.57,1.84]
(5.2) Xuezhikang and simvastatin and conventional therapy versus simvastatin and conventional therapy				
Lin et al. 2009 [27]	1/24	5/24	0.2	[0.03,1.59]

**Table 4:** Analysis of serum lipid level.

Serum lipid level (comparison)	Intervention group		Control group		Weight (%)	MD	95% CI
	Mean	SD	Mean	SD			
*(1) TC (mmol/L)*							
(1.1) Xuezhikang and conventional therapy versus conventional therapy							
Dai et al. 1999 [5]	5.41	0.87	6.54	0.89	11.40	−1.13	[−1.59, −0.67]
Huang et al. 2009 [24]	4.98	0.79	5.99	0.87	13.30	−1.01	[−1.36, −0.66]
Ma and Teng 2005 [29]	5.30	1.30	6.30	1.00	9.00	−1.00	[−1.61, −0.39]
Wang et al. 2004 [6]	4.33	0.96	6.30	0.79	11.10	−1.97	[−2.45, −1.49]
Xu 2005 [39]	5.49	1.12	6.20	0.93	6.60	−0.71	[−1.52,0.10]
Yan 2006 [34]	4.90	0.10	5.50	0.20	17.30	−0.60	[−0.67, −0.53]
Yan and Li 2007 [33]	4.90	0.13	5.93	0.23	17.00	−1.03	[−1.13, −0.93]
Zhou et al. 2003 [38]	4.30	0.54	4.84	0.78	14.30	−0.54	[−0.83, −0.25]
		**Overall (REM, ** *I^2^* = 92%**)**			**100**	−0.97	[−1.24, −0.71]
(1.2) Xuezhikang and conventional therapy versus placebo and conventional therapy							
CCSPS 2005 [4]	4.65	0.67	5.22	0.88	—	−0.57	[−0.61, −0.53]
Yu et al. 2002 [35]	4.10	0.58	6.72	0.85	—	−2.62	[−2.98, −2.26]
(1.3) Xuezhikang and conventional therapy versus statin and conventional therapy							
(1.3.1) Xuezhikang and conventional therapy versus lovastatin and conventional therapy							
Li et al. 2011 [26]	4.57	1.42	5.32	1.72	9.5	−0.75	[−1.52,0.02]
(1.3.2) Xuezhikang and conventional therapy versus simvastatin and conventional therapy							
Huang et al. 2005 [23]	4.62	0.63	4.36	0.60	13.8	0.26	[0.02,0.50]
Jiang and Cai 2001 [25]	5.19	0.90	4.91	0.66	12.8	0.28	[−0.10,0.66]
Lou et al. 2008 [28]	5.4	0.12	5.40	0.11	14.4	0.00	[−0.05,0.05]
	**Subgroup**	**Overall (REM, ** *I^2^* = 69%**)**				0.14	[−0.08,0.35]
(1.3.3) Xuezhikang and conventional therapy versus fluvastatin and conventional therapy							
Gao and Liao 2003 [21]	4.05	0.74	3.63	0.59	13.1	0.42	[0.08,0.76]
(1.3.4) Xuezhikang and conventional therapy versus atorvastatin and conventional therapy							
Shang 2007 [31]	4.65	0.79	4.88	0.85	13.5	−0.23	[−0.51,0.05]
Xu 2005 [39]	5.49	1.12	5.50	0.92	8.8	−0.01	[−0.86,0.84]
Zhang 2011 [37]	4.51	0.38	4.00	3.35	14.1	1.16	[0.99,1.33]
	**Subgroup**	**Overall (REM, ** *I^2^* = 97%**)**				0.33	[−0.77, 1.43]
**After sensitive analysis**	**Subgroup**	**Overall (FEM, ** *I^2^* = 0%**)**				−0.21	[−0.48, 0.06]
	**Total**	**Overall (REM, ** *I^2^* = 96%**)**				0.19	[−0.22,0.59]
**After sensitive analysis**	**Total**	**Overall (REM, ** *I^2^* = 66%**)**				0.02	[−0.032,0.06]
(1.4) Xuezhikang and statin and conventional therapy versus statin and conventional therapy							
(1.4.1) Xuezhikang and simvastatin and conventional therapy versus simvastatin and conventional therapy							
Lin et al. 2009 [27]	4.30	0.71	5.00	0.81	35.6	−0.70	[−1.13, −0.27]
(1.4.2) Xuezhikang and fluvastatin and conventional therapy versus fluvastatin and conventional therapy							
Zhang 2010 [36]	4.60	0.10	5.70	0.24	64.4	−1.10	[−1.19, −1.01]
	**Total**	**Overall (REM,** * I^2^* = 68%**)**				−0.96	[−1.33, −0.58]
(1.5) Xuezhikang and aspirin versus inositol nicotinate and aspirin							
Wang and Xiao 2000 [32]	5.20	0.80	6.00	0.70	—	−1.05	[−1.46, −0.64]
*2. TG (mmol/L)*							
(2.1) Xuezhikang and conventional therapy versus conventional therapy							
Dai et al. 1999 [5]	1.84	0.68	2.30	0.87	5.50	−0.48	[−0.87, −0.05]
Huang et al. 2009 [24]	1.49	0.31	1.97	0.37	44.40	−0.48	[−0.63, −0.33]
Ma and Teng 2005 [29]	1.70	0.40	2.30	0.70	10.50	−0.60	[−0.90, −0.30]
Wang et al. 2004 [6]	1.88	0.5	2.2	0.76	7.70	−0.32	[−0.67,0.03]
Xu 2005 [39]	2.70	0.92	2.52	1.67	0.90	0.18	[−0.87,1.23]
Yan and Li 2007 [33]	1.54	0.10	2.02	0.59	19.10	−0.48	[−0.70, −0.26]
Zhou et al. 2003 [38]	1.20	0.66	1.80	0.61	12.10	−0.60	[−0.88, −0.32]
		**Overall (FEM, ** *I^2^* = 0%**)**			**100%**	−0.49	[−0.58, −0.39]
(2.2) Xuezhikang and conventional therapy versus placebo and conventional therapy							
CCSPS 2005 [4]	1.58	0.78	1.75	0.88	50.80	−0.17	[−0.22, −0.12]
Yu et al. 2002 [35]	2.22	0.71	3.51	0.36	49.20	−1.29	[−1.57, −1.01]
(2.3) Xuezhikang and conventional therapy versus statin and conventional therapy							
(2.3.1) Xuezhikang and conventional therapy versus lovastatin and conventional therapy							
Li et al. 2011 [26]	3.75	1.17	3.82	1.29	1.3	−0.07	[−0.67,0.53]
(2.3.2) Xuezhikang and conventional therapy versus simvastatin and conventional therapy							
Huang et al. 2005 [23]	1.85	0.81	1.92	0.72	5.5	−0.07	[−0.37,0.23]
Jiang and Cai 2001 [25]	1.9	0.72	2.11	0.91	3.5	−0.21	[−0.58,0.16]
Lou et al. 2008 [28]	3.1	0.2	3.2	0.33	35.2	−0.11	[−0.21, 0.00]
	**Subgroup**	**Overall (FEM, ** *I^2^* = 0%**)**			44.3	0.11	[−0.21, −0.00]
(2.3.3) Xuezhikang and conventional therapy versus fluvastatin and conventional therapy							
Gao and Liao 2003 [21]	1.01	0.63	1.42	0.46	6.2	−0.41	[−0.69, −0.13]
(2.3.4) Xuezhikang and conventional therapy versus atorvastatin and conventional therapy							
Shang 2007 [31]	1.61	0.53	1.57	0.55	14.1	0.04	[−0.15,0.23]
Xu 2005 [39]	2.7	0.92	2.22	0.73	1.0	0.48	[−0.21,1.17]
Zhang 2011 [37]	1.64	0.33	1.61	0.21	33.0	0.03	[−0.09,0.15]
	**Subgroup**	**Overall (FEM, ** *I^2^* = 0%**)**			48.1	0.04	[−0.06,0.14]
	**Total**	**Overall (FEM, ** *I^2^* = 45%**)**			100	−0.05	[−0.12,0.02]
(2.4) Xuezhikang and statin and conventional therapy versus statin and conventional therapy							
Zhang 2010 [36]	1.58	0.20	1.85	0.10	—	−0.27	[−0.35, −0.19]
(2.5) Xuezhikang and aspirin versus inositol nicotinate and aspirin							
Wang and Xiao 2000 [32]	1.70	0.90	2.30	0.90	—	−0.60	[−0.95, −0.25]
*(3) LDL-C (mmol/L)*							
(3.1) Xuezhikang and conventional therapy versus conventional therapy							
Dai et al. 1999 [5]	3.42	0.96	3.93	0.81	13.50	−0.51	[−0.97, −0.05]
Huang et al. 2009 [24]	2.88	0.91	3.96	0.96	14.10	−1.08	[−1.48, −0.68]
Wang et al. 2004 [6]	2.21	0.4	3.87	0.56	15.20	−1.66	[−1.92, −1.40]
Xu 2005 [39]	2.82	0.95	3.7	0.95	10.50	−0.88	[−1.63, −0.13]
Yan 2006 [34]	2.89	0.44	2.9	0.6	15.50	−0.01	[−0.23,0.21]
Yan and Li 2007 [33]	2.97	0.10	3.88	0.20	16.20	−0.91	[−0.99, −0.83]
Zhou et al. 2003 [38]	3.22	0.6	3.68	0.71	15.00	−0.46	[−0.75, −0.17]
		**Overall (REM, ** *I^2^* = 94%**)**			100	−0.78	[−1.19, −0.38]
(3.2) Xuezhikang and conventional therapy versus placebo and conventional therapy							
CCSPS 2005 [4]	2.66	0.85	3.23	0.85	50.30	−0.57	[−0.62, −0.52]
Yu et al. 2002 [35]	2.48	0.39	4.30	0.39	49.70	−1.82	[−2.01, −1.63]
(3.3) Xuezhikang and conventional therapy versus statin and conventional therapy							
(3.3.1) Xuezhikang and conventional therapy versus lovastatin and conventional therapy							
Li et al. 2011 [26]	2.45	0.72	3.25	0.84	10.6	−0.80	[−1.18,0.42]
(3.3.2) Xuezhikang and conventional therapy versus simvastatin and conventional therapy							
Huang et al. 2005 [23]	2.68	0.55	2.52	0.49	13.9	0.16	[−0.04,0.36]
Jiang and Cai 2001 [25]	3.1	0.41	2.90	0.90	12.2	0.20	[−0.10,0.50]
Lou et al. 2008 [28]	2.8	0.09	2.9	0.1	15.7	−0.10	[−0.14, −0.06]
	**Subtotal**	**Overall (REM, ** *I^2^* = 79%**)**			41.8	0.06	[−0.17,0.28]
(3.3.3) Xuezhikang and conventional therapy versus fluvastatin and conventional therapy							
Gao and Liao 2003 [21]	2.13	0.58	2.08	0.61	12.2	0.05	[−0.25,0.35]
(3.3.4) Xuezhikang and conventional therapy versus atorvastatin and conventional therapy							
Shang 2007 [31]	2.54	0.56	2.44	0.52	14.2	0.10	[−0.09,0.29]
Xu 2005 [39]	2.82	0.95	2.93	0.52	6.9	−0.11	[−0.74,0.52]
Zhang 2011 [37]	3.04	0.48	2.51	0.32	14.3	0.53	[0.35,0.71]
	**Subtotal**	**Overall (REM, ** *I* ^2^ = 84%**)**			35.4	0.23	[−0.14,0.60]
**After sensitive analysis**	**Subtotal**	**Overall (FEM, ** *I* ^2^ = 0%**)**				0.08	[−0.10,0.26]
	**Total**	**Overall (REM, ** *I* ^2^ = 90%**)**				0.03	[−0.10,0.25]
**After sensitive analysis**	**Total**	**Overall (REM, ** *I* ^2^ = 64%**)**				0.05	[−0.09,0.19]
(3.4) Xuezhikang and statin and conventional therapy versus statin and conventional therapy							
(3.4.1) Xuezhikang and simvastatin and conventional therapy versus simvastatin and conventional therapy							
Lin et al. 2009 [27]	2.10	0.78	2.60	0.80	8.4	−0.50	[−0.95, −0.05]
(3.4.2) Xuezhikang and fluvastatin and conventional therapy versus fluvastatin and conventional therapy							
Zhang 2010 [36]	2.87	0.32	3.30	0.20	91.6	−0.43	[−0.57, −0.29]
	**Total**	**Overall (FEM, ** *I* ^2^ = 0%**)**				−0.44	[−0.57, −0.31]
(3.5) Xuezhikang and aspirin versus inositol nicotinate and aspirin							
Wang and Xiao 2000 [32]	2.70	0.70	3.40	0.90	100	−0.88	[−1.27, −0.48]
*(4) HDL-C (mmol/L)*							
(4.1) Xuezhikang and conventional therapy versus conventional therapy							
Dai et al. 1999 [5]	1.71	0.42	1.04	0.49	14.60	0.67	[−0.43,0.91]
Huang et al. 2009 [24]	1.12	0.3	0.82	0.2	19.50	0.3	[0.19,0.41]
Wang et al. 2004 [6]	1.44	0.38	1.31	0.27	17.00	0.13	[−0.05,0.31]
Xu 2005 [39]	1.67	0.51	1.68	0.75	7.10	−0.01	[−0.51,0.49]
Yan 2006 [34]	1.04	0.10	1.04	0.20	20.60	0.00	[−0.07,0.07]
Yan and Li 2007 [33]	1.09	0.09	0.80	0.07	21.10	0.29	[0.25,0.33]
		**Overall (REM, ** *I* ^2^ = 93%**)**			100	0.24	[0.08,0.40]
(4.2) Xuezhikang and conventional therapy versus placebo and conventional therapy							
CCSPS 2005 [4]	1.24	0.31	1.19	0.31	50.80	0.05	[0.03,0.07]
Yu et al. 2002 [35]	1.45	0.25	0.97	0.19	49.20	0.48	[0.37,0.59]
(4.3) Xuezhikang and conventional therapy versus statin and conventional therapy							
(4.3.1) Xuezhikang and conventional therapy versus lovastatin and conventional therapy							
Li et al. 2011 [26]	1.12	0.38	1.06	0.36	11.4	0.16	[−0.33,0.65]
(4.3.2) Xuezhikang and conventional therapy versus simvastatin and conventional therapy							
Huang et al. 2005 [23]	1.85	0.81	1.92	0.72	6.4	−0.09	[−0.47,0.29]
Jiang and Cai 2001 [25]	1.16	0.17	1.21	0.12	19.0	−0.05	[−0.12,0.02]
Lou et al. 2008 [28]	0.8	0.03	0.9	0.03	21.4	−0.10	[−0.11, −0.09]
		**Overall (FEM, ** *I* ^2^ = 0%**)**				−0.10	[−0.11, −0.09]
(4.3.3) Xuezhikang and conventional therapy versus fluvastatin and conventional therapy							
Gao and Liao 2003 [21]	1.14	0.27	1.30	0.45	11	−0.16	[−0.35,0.03]
(4.3.4) Xuezhikang and conventional therapy versus atorvastatin and conventional therapy							
Shang 2007 [31]	1.45	0.41	1.44	0.33	14.9	0.01	[−0.12,0.14]
Xu 2005 [39]	1.67	0.51	1.53	0.48	3.8	0.14	[−0.27,0.55]
Zhang 2011 [37]	1.09	0.48	1.62	0.27	12.1	−0.53	[−0.70, −0.36]
	**Subtotal**	**Overall (REM, ** *I* ^2^ = 93%**)**			30.9	−0.15	[−0.57,0.28]
**After sensitive analysis**	**Subtotal**	**Overall (FEM, ** *I^2^* = 0%**)**				0.02	[−0.10,0.14]
	**Total**	**Overall (REM, ** *I^2^* = 79%**)**				−0.10	[−0.19, −0.01]
**After sensitive analysis**	**Total**	**Overall (FEM, ** *I^2^* = 35%**)**				−0.10	[−0.11, −0.08]
(4.4) Xuezhikang and fluvastatin and conventional therapy versus fluvastatin and conventional therapy							
Zhang 2010 [36]	0.97	0.28	0.82	0.06	100	0.15	[0.05,0.25]
(4.5) Xuezhikang and aspirin versus inositol nicotinate and aspirin							
Wang and Xiao 2000 [32]	0.95	0.22	0.91	0.25	100	0.17	[−0.21,0.55]

Note: FEM: fixed effects model; REM: random effects model.

**Table 5:** Adverse Events.

Ads/ID	Comparison	Treatment group (*n*/*N*)	Control group (*n*/*N*)	RR	95% CI
*Loss of followup*					
Guan 2010 [22]	Xuezhikang versus simvastatin	16 (72)	15 (64)	0.95	[0.51,1.76]
CCSPS 2005 [4]	Xuezhikang and conventional therapy versus placebo and conventional therapy	37 (2441)	28 (2429)	1.31	[0.81,2.14]
Ma and Teng 2005 [29]	Xuezhikang and conventional therapy versus conventional therapy	1 (29)	No report		

*Intestinal disturbance*					
Guan 2010 [22]	Xuezhikang versus simvastatin	5 (72)	2 (64)	2.22	[0.45,11.06]
Ma and Teng 2005 [29]	Xuezhikang and conventional therapy versus conventional therapy	2 (29)	No report		
Wang et al. 2004 [6]	Xuezhikang and conventional therapy versus conventional therapy	2 (26)	No report		
Jiang and Cai 2001 [25]	Xuezhikang and conventional therapy versus simvastatin and conventional therapy	0 (30)	1 (45)	0.49	[0.02,11.75]
Shang 2007 [31]	Xuezhikang and conventional therapy versus atorvastatin and conventional therapy	No report	1 (65)		
Wang and Xiao 2000 [32]	Xuezhikang and aspirin versus inositol nicotinate and aspirin	5 (65)	2 (57)	2.19	[0.44,10.87]

*Headache*					
Jiang and Cai 2001 [25]	Xuezhikang and conventional therapy versus simvastatin and conventional therapy	1 (30)	0 (45)	4.45	[0.19,105.77]

*Dizziness*					
Guan 2010 [22]	Xuezhikang and conventional therapy versus simvastatin and conventional therapy	0 (72)	10 (64)	0.04	[0.00,0.71]
Jiang and Cai 2001 [25]	Xuezhikang and conventional therapy versus simvastatin and conventional therapy	1 (30)	1 (45)	1.5	[0.10,23.07]
		**Overall (REM, ** *I^2^* = 72%**) **		**0.26 **	[0.01,10.49]

*Skin itech*					
Guan 2010 [22]	Xuezhikang versus simvastatin	0 (72)	3 (64)	0.13	[0.01,2.42]
Wang and Xiao 2000 [32]	Xuezhikang and aspirin versus inositol nicotinate and aspirin	0 (65)	3 (57)	0.13	[0.01,2.38]

*Sexual dysfunction*					
CCSPS 2005 [4]	Xuezhikang and conventional therapy versus placebo and conventional therapy	0 (1996)	5 (1990)	0.09	[0.01,1.64]

*High serum ALT*					
CCSPS 2005 [4]	Xuezhikang and conventional therapy versus placebo and conventional therapy	15 (2441)	22 (2429)	0.68	[0.35,1.30]
Lou et al. 2008 [28]	Xuezhikang and conventional therapy versus simvastatin and conventional therapy	No report	1 (41)		

*High serum CK*					
CCSPS 2005 [4]	Xuezhikang and conventional therapy versus placebo and conventional therapy	0 (2441)	3 (2429)	0.14	[0.01,2.75]

*High serum CR*					
CCSPS 2005 [4]	Xuezhikang and conventional therapy versus placebo and conventional therapy	104 (2441)	89 (2429)	1.16	[0.88,1.53]
*High BUN*					
CCSPS 2005 [4]	Xuezhikang and conventional therapy versus placebo and conventional therapy	124 (2441)	131 (2429)	0.94	[0.74,1.20]
